# Perfluorooctanoic acid induces human Ishikawa endometrial cancer cell migration and invasion through activation of ERK/mTOR signaling

**DOI:** 10.18632/oncotarget.11684

**Published:** 2016-08-29

**Authors:** Zhinan Ma, Xiaoqiu Liu, Fujun Li, Yixong Wang, Yang Xu, Mei Zhang, Xiaoqian Zhang, Xiaoyan Ying, Xuesen Zhang

**Affiliations:** ^1^ Department of Obstetrics and Gynecology, The Second Affiliated Hospital of Nanjing Medical University, Nanjing, China; ^2^ Department of Obstetrics and Gynecology, Yangzhou Maternal and Child Health Hospital, Yangzhou University, Yangzhou, China; ^3^ State Key Laboratory of Reproductive Medicine, Nanjing Medical University, Nanjing, China; ^4^ Key Laboratory of Pathogen Biology of Jiangsu Province, Nanjing Medical University, Nanjing, China; ^5^ Department of Microbiology, Nanjing Medical University, Nanjing, China

**Keywords:** endometrial carcinoma, PFOA, migration and invasion, tumorigenesis, ERK/mTOR

## Abstract

Perfluorooctanoic acid (PFOA) is a common environmental pollutant that has been associated with various diseases, including cancer. We explored the molecular mechanisms underlying PFOA-induced endometrial cancer cell invasion and migration. PFOA treatment enhanced migration and invasion by human Ishikawa endometrial cancer cells, which correlated with decreased E-cadherin expression, a marker of epithelial-mesenchymal transition. PFOA also induced activation of ERK1/2/mTOR signaling. Treatment with rapamycin, an mTOR inhibitor, antagonized the effects of PFOA and reversed the effects of PFOA activation in a xenograft mouse model of endometrial cancer. Consistent with these results, pre-treatment with rapamycin abolished PFOA-induced down-regulation of E-cadherin expression. These results indicate that PFOA is a carcinogen that promotes endometrial cancer cell migration and invasion through activation of ERK/mTOR signaling.

## INTRODUCTION

Perfluorooctanoic acid (PFOA) is a synthetic and persistent organic pollutant found in the environment. Because it is stain- and water-resistant, it is widely used in manufacturing and is found in nearly everything in the environment including drinking water [1, 2]. Therefore, the side effects of PFOA contamination have attracted increasing attention [3, 4]. PFOA has been associated with multiple diseases including cardiovascular disease, peripheral arterial disease, liver damage, birth defects, and cancer [2, 5–7]. Recent studies have shown that PFOA enhanced the invasive behavior of breast and colorectal cancer cells [8, 9]. However, the relationship between PFOA exposure and endometrial cancer has not been elucidated.

Epithelial-mesenchymal transition (EMT) promotes tumor cell motility and invasion [10, 11]. Down-regulation of cell surface markers such as E-cadherin is one of the first alterations during metastatic progression [12, 13]. It normally acts as a tumor suppressor by inhibiting cancer cell invasion and metastasis [14–16]. A variety of signaling pathways are activated during EMT, which can modulate E-cadherin expression in cancer cells [17, 18]. For example, E-cadherin expression can be down-regulated in cancer cells in response to extracellular stimuli through activation of the PI3K/AKT/mTOR and Ras/ERK signaling pathways [19–21]. The phosphatidylinositol-3 kinase (PI3K) pathway is frequently activated in various types of cancer [22, 23]. Upon activation, PI3K phosphorylates AKT, which subsequently phosphorylates several effectors including mammalian target of rapamycin (mTOR). The activation of this pathway plays an important role in the regulation of EMT-associated cell surface markers in cancer cells [24, 25]. Similar to PI3K/AKT/mTOR signaling, the extracellular signal regulated kinase (ERK1/2) signaling pathway exists in various cell types and functions to convert extracellular stimuli into transcriptional programs. Activation of this pathway affects a variety of biological effects, including cell proliferation, differentiation, transformation, and apoptosis [26, 27].

In this study, we evaluated whether PFOA exposure was correlated with E-cadherin expression, cell migration, and invasion in endometrial cancer cells. The molecular mechanisms underlying the response of endometrial cancer cells to PFOA exposure were also investigated.

## RESULTS

### PFOA treatment stimulates Ishikawa cell migration and invasion

**Figure 1:**
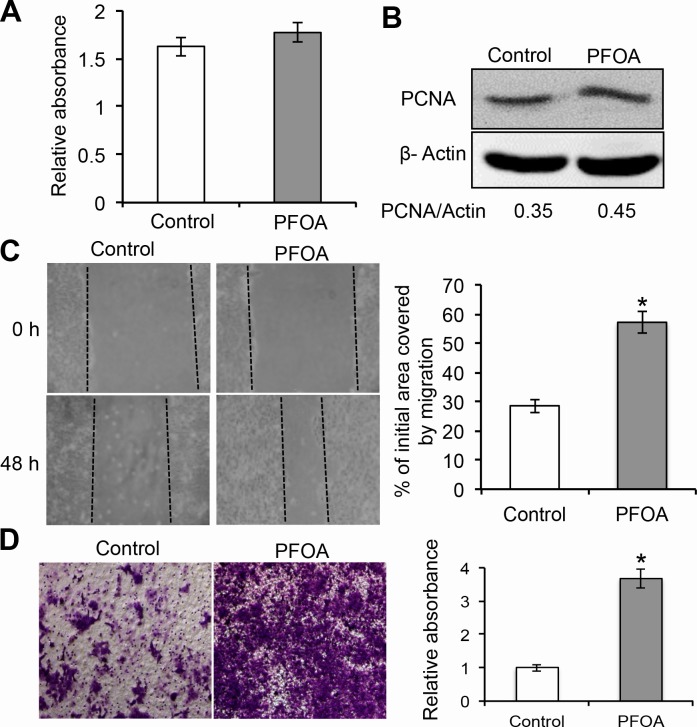
PFOA promotes human Ishikawa endometrial cancer cell migration and invasion **A.** Cell proliferation was assessed using MTT assays after treatment with 50 nM PFOA for 48 hours. **B.** Western blotting to detect PCNA, a marker of cell proliferation. β-Actin was used as a loading control. **C.** Ishikawa cells were cultured to full confluence. Wounding was performed by scratching the cells with a sterile 10 μL pipette tip. After treatment with PFOA, the wound gaps were photographed and measured. **D.** Transwell invasion assays of Ishikawa cells were performed as described in the Materials and Methods. The data are presented as the mean ± SD from three experimental replicates. Control: PBS treatment. *P < 0.05 compared to the mock control.

In order to examine the role of PFOA during human endometrial carcinogenesis, Ishikawa cells were treated with low concentration of PFOA or PBS (control). Previous studies have shown that 50 nM PFOA enhanced the invasive behavior of breast cancer cells [8]. Therefore, we selected a PFOA concentration of 50 nM for all of the following experiments. We first evaluated the effect of PFOA on the proliferation of human Ishikawa endometrial cancer cells. Treatment of Ishikawa cells with PFOA for 48 hours did not affect proliferation (Figure 1A and 1B). We next examined the migration of PFOA-treated Ishikawa cells using wound healing assays. Confluent monolayers of Ishikawa cells were first scratched with 10 μL tips to wound the cells. The cells were then cultured for 48 hours. Changes in the wound healing areas were significantly accelerated in PFOA-treated cells compared to the controls (Figure 1C). The effects of PFOA on Ishikawa cell migration and invasion were further analyzed using a Transwell system. As shown in Figure 1D, PFOA significantly stimulated invasion of the Ishikawa cells through the membranes of the Transwell chambers, which was further confirmed by measuring the optical density (OD) of the invaded cells. These results indicated that PFOA treatment promoted both the migration and invasion of endometrial cancer cells.

### PFOA induces down-regulation of E-cadherin expression in Ishikawa cells

**Figure 2:**
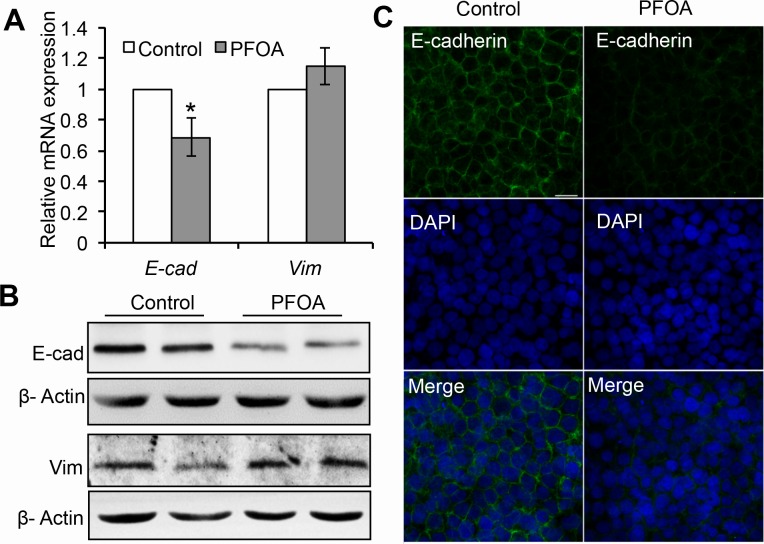
PFOA treatment induces down-regulation of E-cadherin expression in Ishikawa cells **A.** Real-time PCR analysis of *E-cadherin* (*E-cad*) and *vimentin* (*Vim*) expression in serum-starved or PFOA-treated Ishikawa cells. The expression data are normalized to *GAPDH*. The graph represents the mean ± SD (*n* = 3). *P < 0.05. **B.** Reduced expression of E-cadherin (E-cad) detected by western blotting. β-Actin was used as loading control. **C.** Immunofluorescence staining was performed to confirm reduced expression of E-cadherin in response to PFOA (green). Nuclei were stained with DAPI (scale bar: 20 μm).

EMT is the one of the key events in the invasion and migration of many human cancers and is characterized by down-regulation of E-cadherin expression [13, 14]. To investigate whether PFOA treatment promoted EMT, we evaluated the expression of E-cadherin (an epithelial marker) and vimentin (a mesenchymal marker). PFOA treatment resulted in a significant decrease in E-cadherin expression at both the mRNA and protein levels based on real-time PCR and western blotting, respectively (Figure 2A and 2B). Analysis of E-cadherin localization by immunofluorescence demonstrated that PFOA treatment decreased E-cadherin expression (Figure 2C). The levels of vimentin mRNA and protein increased slightly following PFOA treatment, but were not significantly altered. These results raised the possibility that PFOA treatment could promote EMT in endometrial cancer cells.

### PFOA induces activation of mTOR/RPS6 signaling in Ishikawa cells *via* ERK1/2 phosphorylation

**Figure 3:**
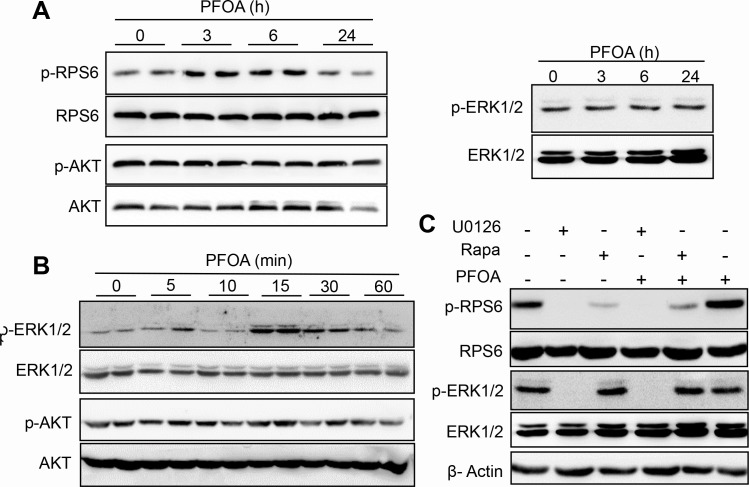
Activation of mTOR/RPS6 signaling in Ishikawa cells after PFOA treatment through ERK1/2 phosphorylation **A.** and **B.** Activation of RPS6, AKT, and ERK after PFOA treatment detected by western blotting. **C.** Ishikawa cells were treated with PFOA, U0126, rapamycin (Rapa), U0126 treatment followed by PFOA, or Rapa treatment followed by PFOA. RPS6 and ERK activation were detected by western blotting. β-Actin was used as a loading control.

The PI3K/AKT/mTOR pathway is frequently altered in endometrial cancer [28, 29]. However, whether PFOA stimulation could activate mTOR signaling was unclear. We therefore assessed the phosphorylation state of RPS6, a downstream effector of mTOR, after PFOA treatment. Interestingly, phosphorylation of RPS6 was observed as early as 3 hours after PFOA treatment. However, the total RPS6 level was not altered. These results suggested that mTOR signaling was activated upon PFOA treatment. Activation of both the PI3K/AKT and ERK signaling pathways could modulate RPS6 during EMT [30, 31]. To determine whether PFOA induced activation of these two signaling pathways in Ishikawa cells, we examined the levels of phosphorylated AKT and ERK1/2 after PFOA treatment. An increase in the levels of phosphorylated AKT or ERK1/2 was not observed 3, 6, or 24 hours after PFOA treatment (Figure 3A). However, a short exposure of Ishikawa cells to PFOA resulted in transient phosphorylation and activation of ERK1/2. ERK2 displayed a reproducible activation pattern with an early phase that occurred approximately 15 minutes after PFOA treatment. The level of activated ERK1/2 gradually decreased to that of the control cells 1 hour after PFOA treatment (Figure 3B). However, AKT activation was not observed within 24 hours of PFOA treatment. These results suggested that PFOA-induced activation of RPS6 may involve the ERK1/2 rather PI3K/AKT pathway.

To confirm the role of ERK1/2 in PFOA-induced RPS6 activation, we treated Ishikawa cells with the MEK1/2 inhibitor U0126 followed by PFOA, which specifically blocked ERK1/2 phosphorylation. Inhibition of ERK1/2 phosphorylation completely blocked RPS6 phosphorylation (undetectable levels) after PFOA stimulation (Figure 3C), indicating that efficient RPS6 activation in Ishikawa cells in response to PFOA requires ERK1/2 signaling. We also confirmed that the mTOR inhibitor rapamycin inhibited PFOA-induced RPS6 phosphorylation, but had no visible effect on ERK1/2 phosphorylation. Thus, PFOA induced activation of mTOR/RPS6 signaling, which did not require AKT and was downstream of ERK1/2 signaling.

### PFOA-induced Ishikawa cell proliferation and migration require activation of mTOR signaling

**Figure 4:**
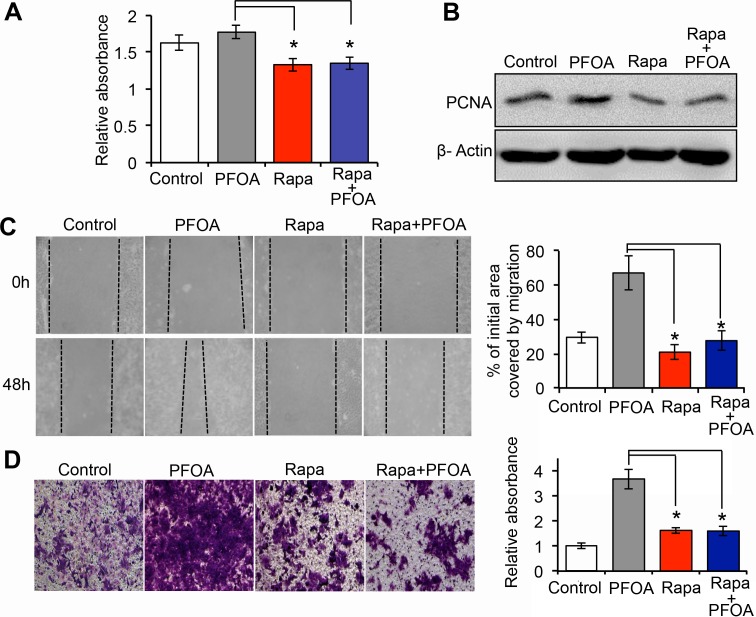
PFOA-induced Ishikawa cell proliferation and migration require activation of mTOR signaling **A.** Cell proliferation was assessed using MTT assays after treatment of the cells with PFOA, rapamycin (Rapa), or Rapa followed by PFOA. **B.** Western blotting to detect PCNA expression, a marker of proliferation. β-Actin was used as a loading control. **C** and **D.** Wound healing and Transwell invasion assays in Ishikawa cells were performed after PFOA, rapamycin (Rapa), or Rapa followed by PFOA treatment. The procedures were performed as described in Figure 1C and 1D. Control: PBS treatment. *P < 0.05.

To investigate the role of mTOR signaling in PFOA-induced Ishikawa cell growth, we first examined the effect of pre-treating cells with rapamycin prior to PFOA exposure on cell proliferation. Rapamycin treatment suppressed cell proliferation. This effect was not reversed by the addition of PFOA, suggesting that PFOA-induced Ishikawa cell proliferation required mTOR signaling (Figure 4A and 4B). We demonstrated that treatment of Ishikawa cells with PFOA promoted cell motility and invasion (Figure 1C and 1D). To determine whether the Ishikawa cell response to PFOA was dependent on the mTOR pathway, we analyzed the effects of rapamycin on the migration and invasion of PFOA-treated cells. Using a specific mTOR inhibitor, we found that PFOA-enhanced cell migration was reduced to approximately the same level as the control in wound healing assays (Figure 4C). Rapamycin treatment also reduced the invasive capacity of PFOA-treated cells. Finally, exposure of rapamycin-treated Ishikawa cells to PFOA did not alter invasion compared to control cells (Figure 4D).

### PFOA-induced down-regulation of E-cadherin expression in Ishikawa cells requires activation of mTOR signaling

**Figure 5:**
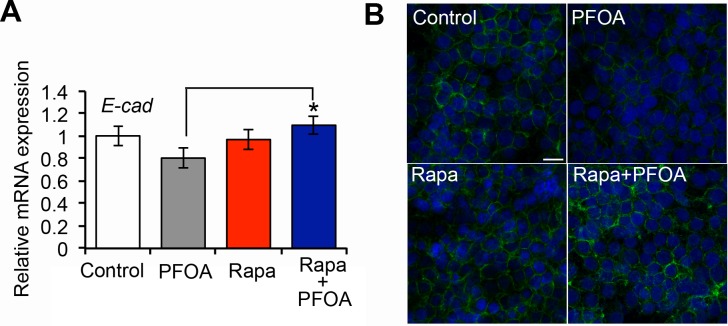
Decreased E-cadherin expression in Ishikawa cells after PFOA stimulation requires activation of mTOR signaling **A.** Real-time PCR analysis of the *E-cadherin* (*E-cad*) expression in Ishikawa cells after PFOA-, rapamycin (Rapa), or Rapa followed by PFOA treatment. Serum-starved Ishikawa cells were used as a control. The expression data were normalized to *GAPDH*. The graph represents the mean ± SD (*n* = 3). *P < 0.05. **B.** Immunofluorescence staining of E-cadherin (green) in Ishikawa cells after PFOA, rapamycin (Rapa), or Rapa followed by PFOA treatment. Nuclei were stained with DAPI (scale bar: 20 μm).

Because the mTOR inhibitor could reduce PFOA-induced cell proliferation, migration, and invasion, we examined whether rapamycin treatment could abrogate the inhibitory effect of PFOA on E-cadherin expression. Ishikawa cells were pretreated with 250 nM rapamycin for 3 hours. PFOA was then added and the cells incubated for 48 hours. We then analyzed the cells by real-time PCR and immunofluorescence staining. Our results showed that the PFOA-induced reduction in E-cadherin expression was reversed by mTOR inhibition (E-cadherin levels returned to levels that were similar to controls) (Figure 5A and 5B). These data suggested that PFOA could induce EMT in Ishikawa cells, and that this effect was reversed by inhibition of mTOR.

### PFOA promotes tumorigenesis *in vivo via* activation of the mTOR signaling pathway

**Figure 6:**
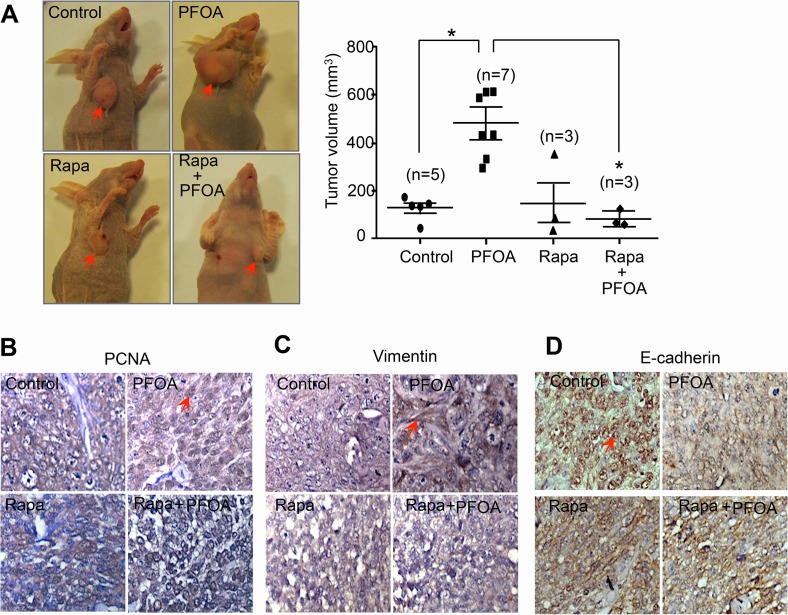
PFOA promotes tumorigenesis *in vivo via* activation of mTOR signaling **A.** Xenograft mice were established as described in the Materials and Methods. Tumors were collected 5 weeks after cancer cell injection. Representative images of the tumors and the average tumor volumes for the control-, PFOA-, rapamycin (Rapa)-, and Rapa followed by PFOA-treated groups are shown. *P < 0.05. **B.-D.** PCNA, vimentin, and E-cadherin expression were analyzed in tumor tissues by immunohistochemistry. Representative images are shown. The arrow indicates positive staining. Magnification, ×40.

To further investigate the stimulation effect of PFOA on EMT *in vivo*, we assessed whether PFOA-mediated activation of mTOR signaling contributed to tumorigenesis in a xenograft mouse model of endometrial cancer. Nude mice were inoculated with Ishikawa cells and tumor volume was analyzed in control and PFOA-treated mice. The PFOA-treated mice all exhibited significantly larger tumors than control mice (Figure 6A). To test whether mTOR signaling was associated with PFOA-induced EMT, rapamycin was administered to the nude mice along with PFOA. Rapamycin treatment antagonized the effect of PFOA and the tumor volumes in these mice were comparable to those of the control mice. Immunohistochemical analysis revealed stronger PCNA (Figure 6B) and vimentin (Figure 6C) staining in the PFOA group compared to the control group. Interestingly, rapamycin treatment abolished the effects of PFOA. In contrast, E-cadherin expression was reduced in the PFOA-treated group compared to the controls, and the signals were comparable to the control levels after rapamycin treatment (Figure 6D).

## DISCUSSION

PFOA exposure was previously shown to promote both breast and colorectal cancer cell invasion [8, 9]. However, the mechanisms underlying the association between PFOA exposure and endometrial carcinoma have not been elucidated. We demonstrated that PFOA could enhance human Ishikawa endometrial cancer cell migration and invasion *in vitro* (Figure 1 and 4) and tumorigenesis *in vivo* (Figure 6). Thus, PFOA likely functions as a carcinogen that promotes endometrial cancer. We determined that PFOA treatment did not significantly affect Ishikawa cell proliferation. This result is consistent with those of previous studies, which showed that treatment of various cancer cells with lower concentrations of PFOA did not affect cell viability [8, 9]. We also demonstrated that PFOA treatment decreased E-cadherin expression in Ishikawa cells (Figure 2 and 4), suggesting that PFOA could promote EMT. PFOA-induced EMT required activation of the mTOR/RPS6 signaling pathway, which could be reversed by rapamycin treatment (Figure 4–6). These results provided evidence for an active role of mTOR signaling in PFOA-induced Ishikawa cell migration and invasion *in vitro* and tumorigenesis *in vivo*.

Inhibition of PI3K/AKT/mTOR resulted in down-regulation of the expression of EMT markers in cancer cells [32, 33]. Therefore, the PI3K/AKT pathway is a promising target for endometrial cancer therapy. Interestingly, although RPS6 was activated after PFOA treatment, PI3K/AKT signaling may not be responsible for the effects of PFOA in Ishikawa cells. Instead, PFOA-induced mTOR/RPS6 activation required ERK1/2 signaling (Figure 3). There is evidence that ERK1/2 can activate mTOR signaling. For example, phosphatidic acid, a lipid second messenger, acts through parallel upstream ERK signaling to activate the mTOR pathway [33]. The Ras/MAPK pathway is upstream of mTOR signaling, suggesting that ERK1/2 may modulate mTOR signaling and contribute to tumor progression [34]. We also examined the effects of PFOA on ECC1 endometrial cancer cell proliferation, migration, and invasion. PFOA treatment slightly increased cell proliferation. Rapamycin could reduce PFOA-induced proliferation (Figure S1A). In Transwell invasion and wound healing assays, PFOA-induced cell migration and invasion were significantly reduced to approximately the same level as the control by rapamycin (Figure S1B and S1C). PFOA also inhibited the E-cadherin expression as determined by western blotting and immunofluorescence assays. However, rapamycin treatment abolished PFOA-induced down-regulation of E-cadherin and up-regulation of vimentin expressions (Figure S2). These results indicated that PFOA treatment induced an EMT phenotype similar to that observed in Ishikawa cells, which also required the activation of mTOR signaling.

Overall, our results suggest that PFOA regulates endometrial carcinoma cell migration and invasion through activation of ERK/mTOR signaling, which could promote tumorigenesis. A detailed mechanistic understanding of the oncogenic activities of PFOA is important from an environmental health standpoint, and may also be useful for endometrial cancer prevention and/or treatment.

## MATERIALS AND METHODS

### Cell culture

Ishikawa and ECC1 cells were maintained in RPMI-1640 media (Gibco, USA) supplemented with 10% newborn calf serum (Gibco, USA) and 10% fetal bovine serum (Gibco, USA), at 37°C in a humidified 5% CO2 incubator. The cells were serum starved for 48 hours and subsequently stimulated with 50 nM PFOA (Sigma-Aldrich, USA). Where indicated, cells were pre-treated with the MAP kinase inhibitor U0126 at a concentration of 10 μM or the mTOR inhibitor rapamycin at 250 nM for 24 hours before the addition of PFOA.

### MTT assays

Cell proliferation was measured using 3-(4, 5-dimethylthiazol-2-yl)-2,5-diphenyl-tetrazolium bromide (MTT) assays. The cells were seeded in six wells of each 96-well culture plate at a density of 2 × 10^3^ cells/well and incubated at 37°C for 24 hours. The cells were cultured in serum-free RPMI-1640 for 2 days, and then incubated with PFOA for an additional 48 hours (or pre-treated with rapamycin and then incubated with PFOA). A total of 20 μL of MTT (5 mg/mL in phosphate-buffered saline, PBS) was added to each well and the plates incubated in the dark at 37°C for 4 hours. Culture media was discarded and 200 μL of dimethyl sulfoxide (DMSO) added to dissolve the formazan crystals at room temperature for 10 min. The absorbance was measured at 490 nm using a FLUOstar Omega-BMG plate reader. Cell viability = OD_490 nm_ of the treatment/OD_490 nm_ of the control.

### Wound healing assays

Cell migration was assessed using wound-healing assays. The cells were seeded in six-well plates and grown to full confluence in complete media (three wells per condition). The monolayer was scratched with a 10 μL pipette tip and then washed twice with serum-free RPMI-1640 to remove the detached cells. The cells were incubated for additional 48 hours in serum free RPMI-1640 in the presence of 0 or 50 nM PFOA, rapamycin alone, or rapamycin (added 24 hours before PFOA stimulation) and PFOA. The wounded areas were observed and imaged by microscopy. Differences in cell migration were quantified by comparing the wound healing areas after 48 hours in at least four fields using ImageJ (NIH, USA). Data from three independent experiments were used to calculate the final data.

### Transwell invasion assays

Transwell invasion assays were performed in 24-well plates with 8 μm pore size chamber inserts (Corning, NY, USA) according to the manufacturer's protocols. Briefly, the upper surface of the filter was coated with 50 μL of Matrigel diluted 1:3 in serum-free RPMI-1640. Approximately 4 × 10^4^ cells were added to the upper chambers of the Matrigel-coated Transwells and cultured in serum-free RPMI-1640. Treatment of the cells with PFOA exposure and the inhibitors was performed as described above. The lower compartment of the Transwell chamber was filled with 600 μL complete media. Cells on the lower surface were then fixed with 4% paraformaldehyde, stained with 0.1% crystal violet, and imaged (three independent fields per well) under a light microscope at a magnification of x40. Finally, the cells were extracted with 33% acetic acid and analyzed with a standard microplate reader (OD at 570 nm).

### Western blotting

The cells were washed twice with cold PBS and then harvested for Western blotting. Cells were lysed in cold radioimmunoprecipitation assay (RIPA) buffer containing protease inhibitors for 30 min. The lysates were then centrifuged and the supernatants collected. Approximately 40 μg of total protein was denatured and separated by 10% SDS-PAGE, and then transferred to a nitrocellulose membrane. The membranes were blocked with 5% non-fat milk in Tris-buffered saline containing 0.1% Tween-20 (TBST) for 2 hours at room temperature. The membranes were then incubated with the following primary antibodies overnight at 4°C: PCNA (Abcam, USA), E-cadherin (Cell Signaling Technology, USA), Vimentin (Santa Cruz Biotechnology, USA), p-RPS-6, RPS-6, p-AKT, AKT, p-ERK1/2, and ERK1/2 (Cell Signaling Technology, USA). β-actin (Santa Cruz Biotechnology, USA) was used as a loading control. The membranes were washed five time with TBST and then incubated with horseradish peroxidase-conjugated secondary antibodies for 1 hour at room temperature. The signals were visualized using an Enhanced Chemiluminescence Detection Kit (Pierce Biotechnology, USA).

### Quantitative real-time PCR

Total RNA was isolated from cells using the Qiagen RNeasy Mini Kit in combination with on-column DNase treatment (Applied Biosystems, USA). A High Capacity RNA-to-cDNA Kit (Applied Biosystems, USA) was used to synthesize the first strand of cDNA. Quantitative real-time PCR was performed using the Power SYBR Green PCR Master Mix (Applied Biosystems, USA) with gene-specific primers. The primers were the following: *E-cadherin*, 5′-TGG AGG AAT TCT TGC TTT GC-3′ (F), 5′-CGC TCT CCT CCG AAG AAA C-3′ (R); *vimentin*, 5′-GGC TCG TCA CCT TCG TGA AT-3′ (F), 5′-GAG AAA TCC TGC TCT CCT CGC-3′ (R); and *GAPDH*, 5′-ACC CAT CAC CAT CTT CCA GGA G-3′ (F), 5′-GAA GGG GCG GAG ATG ATG AC-3′ (R). All target gene transcripts were normalized to *GAPDH*, and the relative fold change in expression calculated using the 2^−ΔΔCT^ method.

### Immunofluorescence

Cells were grown on glass slides in 24-well plates. PFOA exposure and inhibitor treatment were performed as described above. After washing with PBS, the cells were fixed with 4% paraformaldehyde for 30 min and then permeabilized with 0.1% Triton X-100 for 10 min. The cells were blocked with 10% goat serum/1% BSA in PBS for 1 hour at room temperature. The cells were then incubated with an anti-E-cadherin primary antibody overnight at 4°C, and a Fluor 488-conjugated goat anti-rabbit secondary antibody (Invitrogen, USA). The nuclei were stained with DAPI (Vector Laboratories, UK). Representative images were collected using a LSM 510 laser scanning confocal microscope (Carl Zeiss, Germany).

### Xenograft mouse model

Female BALB/c nude mice (6-week-old) were purchased from the Shanghai Laboratory Animal Center (Chinese Academy of Sciences, Shanghai, China) and maintained in a special pathogen-free (SPF) environment. All procedures were reviewed and approved by the Institutional Animal Care and Use Committee of Nanjing Medical University. Ishikawa cells (1 × 10^7^) were suspended in 200 μL of cold PBS and injected subcutaneously into the left upper flank of the mice. The mice (*n* = 18) were randomly divided into control and experimental groups. The number of mice in each group is shown in Figure 6A. PFOA was administered p.o. daily for 3 weeks at a dose of 20 mg/kg/day starting on the same day as the cell injection. Sterile water was administered to mice in the control group. Rapamycin was injected intraperitoneally at a dose of 2 mg/kg every 2 days for a total of 5 weeks. Tumor diameters were measured with digital calipers, and the tumor volumes in mm^3^ calculated using the following formula: Volume = 0.5x (Width)^2^ × Length.

### Immunohistochemistry

Mouse tumor tissue was fixed in 10% buffered formalin, embedded in paraffin, and then sectioned for immunohistochemical analysis. The samples were deparaffinized and rehydrated, and then incubated for 10 min in 3% H_2_O_2_ to quench endogenous peroxidase activity. Sections were then heated to retrieve the antigen. Immunohistochemical analyses were performed using a Histostain Kit (Invitrogen, USA) with antibodies against PCNA, vimentin, and E-cadherin overnight at 4°C.

### Statistical analysis

All experiments were performed at least three times. The data are presented as the mean ± standard deviation (SD). Statistical significance was assessed using Student's *t*-tests. A *P* < 0.05 was considered significant (denoted by an asterisk).

## SUPPLEMENTARY MATERIAL FIGURES


