# Supplementary material for: Perfluorooctanoic acid induces human Ishikawa endometrial cancer cell migration and invasion through activation of ERK/mTOR signaling
Source: Oncotarget. 2016 Aug 29;7(41):66558–68. doi: 10.18632/oncotarget.11684 (PMC5341820; doi:10.18632/oncotarget.11684)
Supplement: Supplementary file 1 [file oncotarget-07-66558-s001.pdf]

Perfluorooctanoic acid induces human Ishikawa endometrial cancer cell migration and invasion through activation of ERK/mTOR signaling

Supplementary Material

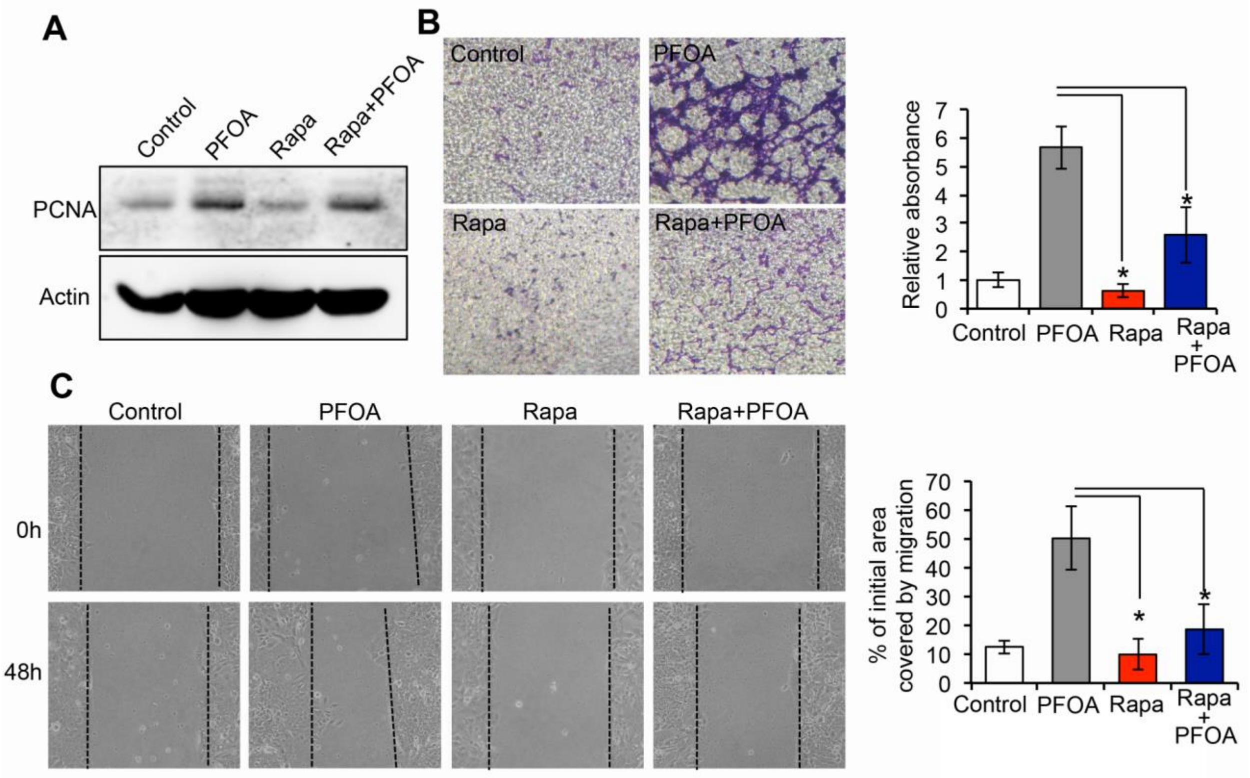

Figure S1. PFOA-induced ECC1 cell proliferation and migration requires activation of mTOR signaling. A. Western blotting to detect PCNA expression, a marker of cell proliferation, in control- (PBS), PFOA-, rapamycin (Rapa)-, and Rapa followed by PFOA-treated cells. β-Actin was used as a loading control. **B.** and **C.** Transwell invasion and wound healing assays were performed under PFOA, Rapa, or Rapa followed by PFOA, treatment. Control: PBS treatment. *$P < 0.05$.

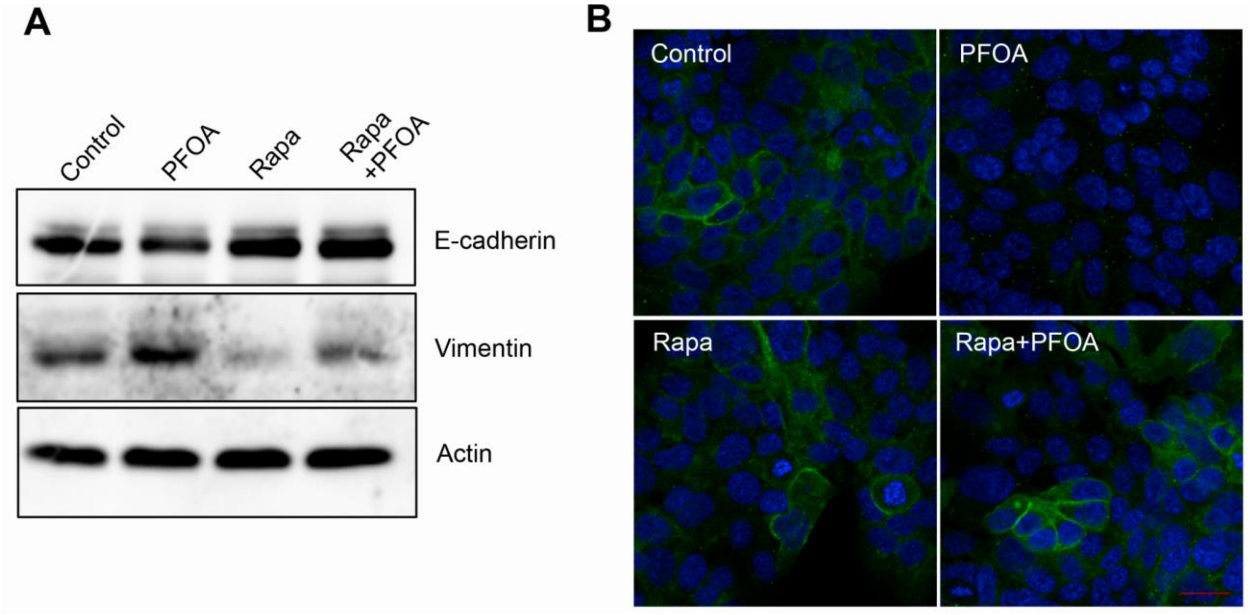

Figure S2. Decreased E-cadherin expression in ECC1 cells after PFOA treatment requires activation of mTOR signaling. A. Western blotting to detect E-cadherin and vimentin expression in control- (PBS), PFOA-, Rapa-, or Rapa followed by PFOA-treated cells. β-Actin was used as a loading control. **B.** Immunofluorescence staining of E-cadherin (green) in PFOA-, Rapa-, or Rapa followed by PFOA-treated cells. Nuclei were stained with DAPI (scale bar: 20 μm).
